# Exploring the Inherent Variability of Economically Fabricated ZnO Devices Towards Physical Unclonable Functions for Secure Authentication

**DOI:** 10.3390/mi16060627

**Published:** 2025-05-26

**Authors:** Savvas Ermeidis, Dimitrios Tassis, George P. Papageorgiou, Stavros G. Stavrinides, Eleni Makarona

**Affiliations:** 1 Department of Condensed Matter and Materials Physics, School of Physics, Aristotle University of Thessaloniki, 541 24 Thessaloniki, Greece; sermeid@auth.gr; 2 Institute of Nanoscience and Nanotechnology, NCSR “Demokritos”, Agia Paraskevi, 153 41 Athens, Greece; g.p.papageorgiou@inn.demokritos.gr; 3 Physics Department, Democritus University of Thrace, St. Lucas, 654 03 Kavala, Greece; sstavrin@physics.duth.gr

**Keywords:** physical unclonable functions (PUFs), authentication elements, photodiodes, homojunctions, ZnO nanostructures, Li doping, hydrothermal growth

## Abstract

Meeting the rising need for secure authentication in IoT and Industry 4.0, this work presents chemically synthesized ZnO nanostructured homojunctions as powerful and scalable physical unclonable functions (PUFs). By leveraging intrinsic variability from Li doping and the stochastic hydrothermal growth process, we systematically identified electrical parameters offering outstanding variability, stability, and reproducibility. ZnO devices outperform commercial diodes by delivering richer parameter diversity, lower costs, and superior environmental sustainability. Pushing beyond traditional approaches, we introduce multi-level quantization for boosted accuracy and entropy, demonstrate the normal distribution of challenge candidate parameters to support a novel method under development, and extract multiple parameters (8–10) per device instead of relying on a single-bit output. Parameter optimization and selection are performed upfront through a rigorous assessment of variability and inter-correlation, maximizing uniqueness and reliability. Thanks to their strong scalability and eco-friendliness, ZnO-based homojunctions emerge as a dynamic, future-proof platform for building low-cost, high-security, and sustainable digital identity systems.

## 1. Introduction

Data security is a major concern in today’s technological landscape, particularly within frameworks like the Internet of Things (IoT), big data, and Industry 4.0 ecosystems [1,2]. The rapid advancements in computational power, combined with the disruptive potential of quantum computing, underscore the need for robust encryption, identification, and authentication methods. Current approaches relying on complex algorithms or expanding cryptographic key sizes create a feedback loop, increasing computational capability to break security systems and corresponding growth in security measures, which drives further computational demands [3].

A promising alternative focuses on utilizing hardware-based physical properties to develop security solutions. This method has gained significant attention due to the rise of IoT and the widespread use of electronic devices such as sensors and actuators. By leveraging the distinct physical characteristics of electronic components—differences inherent to their fabrication—it becomes possible to create unique identifiers. These identifiers, known as physical unclonable functions (PUFs), are highly secure due to their uniqueness and reproducibility [4]. The unclonability of PUFs is one of their most significant advantages, as the inherent variations in manufacturing make it virtually impossible to duplicate a PUF, even with access to the same device model. Furthermore, PUFs are inherently tamper-resistant. Since they rely on the physical characteristics of the hardware, any attempt to clone or probe the device typically results in physical damage, adding another layer of protection [5]. Additionally, PUFs offer low overhead for integration, as they require minimal power, area, and cost to be incorporated into electronic devices. This makes them an ideal solution for low-cost or resource-constrained applications like IoT devices [6].

However, the implementation of PUFs also presents challenges. Noise and stability can be significant issues, as environmental factors such as temperature and voltage fluctuations may affect the response of PUFs. Over time, these changes can degrade the reliability of the responses, making error correction–retraining techniques necessary for maintaining the effectiveness of PUFs in real-world applications. Furthermore, PUFs face a limited lifetime. As they depend on the physical properties of the hardware, the prolonged use or aging of the components can reduce their reliability, especially in high-demand systems [7]. Despite their unique characteristics, PUFs are not immune to security attacks. Advanced attackers can use modeling attacks to predict a PUF’s response by observing its behavior over time, although robust designs can mitigate these risks [8].

Successfully tackling these issues could have immediate applications in critical sectors. Enhanced device authentication can safeguard sensitive infrastructures, such as data centers, public transport systems, and smart city control hubs. These measures could prevent cyberattacks targeting critical systems, including energy grids, waste management, and logistics networks.

To maximize impact, digital authentication solutions based on PUFs should be compatible with current and future manufacturing techniques for integrated circuits and nanoelectronics. This would ensure they are an integral part of any sui generis application, minimize costs, and provide the adaptability needed for diverse applications. Furthermore, it is essential to devise new materials and fabrication processes that align with the principles of a sustainable and low-carbon-footprint semiconductor industry. By prioritizing the use of abundant and environmentally benign materials, these solutions can reduce reliance on rare and hazardous elements that harm the environment. Additionally, incorporating user-friendly and non-toxic materials in both the design and manufacturing processes ensures broader applicability and minimizes ecological impact. These advancements would enable the development of authentication technologies that not only meet the technical demands of modern electronic systems but also contribute to a more sustainable, accessible, and environmentally responsible technological landscape [9].

In this study, we propose the use of chemically produced ZnO nanostructured homojunctions as authentication elements, capitalizing on their inherent variations induced via chemical doping with different concentrations of Li and the size-dependent variations arising from the stochastic nature of the chemical growth. This approach is motivated by three key factors:

(i) ZnO is an abundant and low-cost material known for its exceptional versatility, encompassing a broad spectrum of electrical, optical, and piezoelectric properties [10,11,12,13]. Its wide bandgap and high exciton binding energy make it a strong candidate for optoelectronic applications, while its biocompatibility and chemical stability extend its utility to biomedical and environmental technologies [14,15,16]. Additionally, ZnO exhibits excellent thermal and mechanical robustness, making it suitable for a variety of conditions and applications (e.g., [17,18,19,20,21,22,23]). This multifaceted nature underpins its relevance in numerous cutting-edge technological fields, further justifying its selection for this study. Importantly, the use of ZnO aligns with efforts to transition toward a sustainable semiconductor industry by relying on abundant and environmentally benign materials.
(ii) The hydrothermal synthesis of metal oxide nanostructures is not only economical, rapid, and applicable to a large variety of substrates (from standard silicon wafers all the way to flexible substrates, fabrics, and wood, e.g., [13,16,24]) but also eliminates the need for expensive equipment or infrastructure [25]. Furthermore, the morphology and properties of the resulting nanostructures can be finely tuned by adjusting simple synthesis parameters such as temperature, precursor salt concentration, dopant concentration, or pH. This flexibility provides an almost limitless spectrum of variability, making it highly suitable for PUFs. Additionally, hydrothermal synthesis is inherently more sustainable compared to conventional fabrication methods, consuming fewer resources and generating a lower environmental impact. The ability to create such structures with minimal energy and without reliance on toxic or hazardous chemicals ensures that the process remains environmentally friendly and suitable for user-friendly applications.
(iii) Hydrothermal synthesis is compatible with standard micro- and nanofabrication techniques [15,26,27,28,29,30], enabling the production of devices that are both easy and cost-effective to fabricate, even at a low-carbon-footprint mass-production scale.

Together, these principles highlight the potential for utilizing materials and fabrication processes that prioritize sustainability, cost-efficiency, and mass production while maintaining functionality.

The study is organized into three consecutive parts to ensure a logical progression from concept to implementation. The first part introduces the general framework of physical unclonable functions (PUFs) and their application as secure authentication elements, with an emphasis on the inherent variability of diodes (Section 2.1). This is followed by a detailed description of the devices used in this study: commercially available semiconductor diodes (Section 2.2), and ZnO-based devices fabricated via hydrothermal synthesis (Section 2.3).

The second part of the study focuses on demonstrating the feasibility of using semiconductor devices as authentication elements. Commercial diodes are employed as a proof of concept to validate the approach (Section 3.1). The third part explores the potential of ZnO-based homojunctions to generate robust and secure PUFs through their intrinsic variability (Section 3.2), culminating in a comparison of commercial diodes and ZnO-based devices in terms of cost, environmental impact, and security potential (Section 3.2).

A key focus of the study is demonstrating the variability that makes ZnO-based devices uniquely suited to PUF generation. This variability arises from two primary factors: variations in their doping profiles, which affect their electrical characteristics, and differences in electrode surface area, resulting from the stochastic nature of the hydrothermal nanostructure growth process. Owing to this variability, several key innovations are introduced, including multi-level quantization to enhance accuracy and entropy, and proof of normal distribution for challenge candidate parameters, forming the basis for an emerging method. Unlike conventional approaches that rely on a single parameter per device [31], multiple parameters (8–10) are extracted, with optimization and selection performed prior to PUF construction based on variability and correlation analysis. These innovations, combined with strong scalability, position the approach as a promising solution for high-entropy authentication. The use of hydrothermally grown ZnO nanostructures exemplifies how “humble” materials, sub-optimally performing devices, and alternative fabrication techniques can enable secure, cost-effective solutions. This work highlights the untapped potential of these materials and methods, serving as a proof of concept for future advancements in sustainable, high-security authentication technologies.

## 2. Materials and Methods

### 2.1. PUFs and Diodes—General Framework

The realization of physical unclonable functions (PUFs) hinges on materials or devices exhibiting unique and reproducible characteristics. Diodes and light-emitting diodes (LEDs) emerge as highly promising candidates for PUF implementation due to their inherent variability and the abundance of parameters that can be exploited to establish digital identities. Key parameters include the saturation current (*I_s_*), ideality factor (*η*), turn-on voltage (*V_ον_*), and resistances (both series, *R_s_*, and parallel, *R_p_*), among others [6,9,32]. Each of these characteristics offers a distinct fingerprint, enabling the creation of secure challenge–response schemes that underpin dynamic authentication protocols [3,9,32].

**Figure 1:**
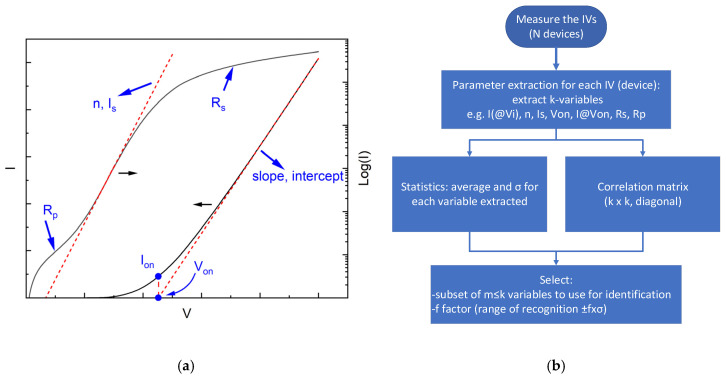
(**a**) Typical forward I-V characteristics of a diode and suggested parameters to extract (n, *I_s_*, *V_on_*, *I_on_*) from each region. (**b**) Flowchart describing the steps followed from the start (measuring the I-Vs of N similar devices) until the end of the workflow. The result is to select the minimum efficient subset of recognition parameters and maximum acceptable recognition limits—in terms of the standard deviation σ-, necessary for a successful device identification within the ensemble, or other possible devices under test.

Figure 1a illustrates a typical current–voltage (I–V) response of a diode, highlighting its distinct operating regions, each of which offers parameters critical for PUF generation. In the forward bias regime, parameters such as the ideality factor (*η*) and saturation current (*I_s_*) are derived from the exponential region of the I-V curve. At higher forward voltages, the series resistance (*R_s_*) becomes prominent, reflecting the diode’s bulk and contact resistances. Conversely, in the low-voltage region, the parallel resistance (*R_p_*) is extracted, accounting for leakage currents and shunting paths. Another critical parameter is the turn-on voltage (*V_on_*), which varies from device to device, providing a unique identifier. Also, the current *I_on_* corresponding to *V_on_*, i.e., *I_on_* = *I*(*V_on_*), can similarly be of interest. Additionally, currents at specific biases *I*(*V_i_*) and series resistances calculated at specific biases *R_s_*(*V_i_*) can be extracted and examined as unique identifiers.

A reverse bias operation further expands the range of exploitable parameters, introducing characteristics such as breakdown voltage and reverse leakage current. These additional features amplify the potential for uniqueness, ensuring that even devices fabricated under identical conditions can exhibit distinguishable characteristics.

The general framework for discovering the unique identifiers for each type of device (in our case diodes) is shown in Figure 1b. Briefly, after acquiring the I-V characteristics of *N* devices, one analyzes each one in order to extract *k* parameters of interest from each one ending with a matrix of data having *N* rows and *k* columns (*N* × *k*). For each column, one estimates the mean value and the standard deviation, σ. Subsequently, for any two columns, one estimates their correlation coefficient, *r_xy_*, in order to find hidden inherent correlations according to the following well-known formula:

(1) $$r_{x,y}=\frac{N\sum \left(xy\right)-(\sum x)(\sum y)}{\sqrt{N\sum x^{2}-{\left(\sum x\right)}^{2}}\sqrt{N\sum y^{2}-{\left(\sum y\right)}^{2}}},$$

where *x* and *y* denote the values from any pair of two parameters, *x*,*y* ∈ *k*.

Among the *k* parameters, the focus is on those exhibiting substantial variability while remaining as uncorrelated as possible. One must assess which *m* parameters (where *m* ≤ *k*) display the highest degree of variability while exhibiting low correlation. A correlation coefficient, *r*, between 0.3 and 0.4 is marginally acceptable for use as a unique identifier, while an *r* value below 0.3 is preferable. Using these criteria, one can test parameter combinations to identify the optimal subset that ensures efficient and secure device recognition. This can be readily done by depicting the correlation coefficients in the form of a *k* × *k* matrix with each element, *r_ij_*, being the correlation coefficient of parameters *k_i_*, *k_j_* (*i* ≠ *j*, *i*,*j* ∈ [1, *k*]). Obviously, all diagonal elements, *r_ii_*, are equal to 1 (being the correlation of each parameter to itself). Additionally, the individual standard deviation, *σ*, of each parameter is valuable and will be utilized in the recognition procedure, as further explained in Section 3.2.

By systematically selecting and analyzing these parameters, one can establish a framework for generating reliable and distinctive device fingerprints. This approach underpins the robustness of diodes as PUF elements, enabling the generation of secure, unclonable responses crucial for modern authentication systems. Leveraging these characteristics, diodes can effectively meet the stringent requirements of PUF applications, demonstrating their practicality and effectiveness in secure digital identity frameworks.

### 2.2. Commercial Diodes

In order to demonstrate the general concept of the suitability of diodes as PUF generators as described above, commercial LEDs were used. In total, 49 white, 100 yellow, 100 red, 100 blue, and 100 green (5 mm) LEDs were tested. Each batch of colors was of the same model and manufacturer. Each diode was given a unique ID (number) to enable back-tracking and verification.

### 2.3. Hydrothermally Produced ZnO Homojunction Devices

The ZnO homojunction devices were fabricated following a two-step solution-based process, which has already been employed in previous works and has been proven to be compatible with standard micro/nanofabrication techniques [15,27,28,29,30,33]. All chemicals were used as obtained without any further purification.

**Figure 2:**
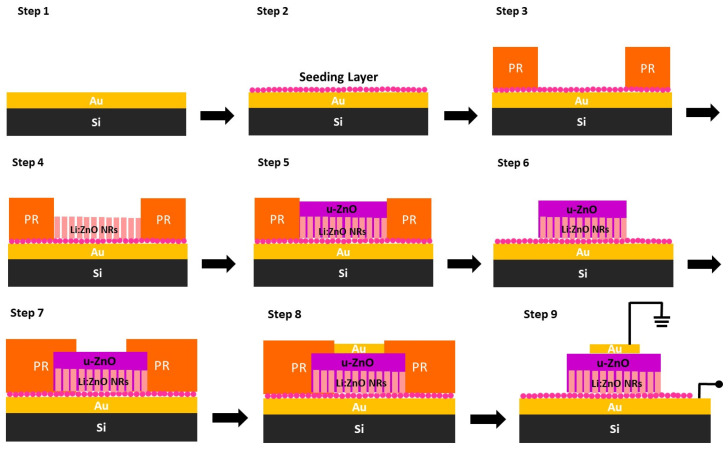
Schematic representation of the fabrication process flow for the ZnO homojunction devices.

**Figure 3:**
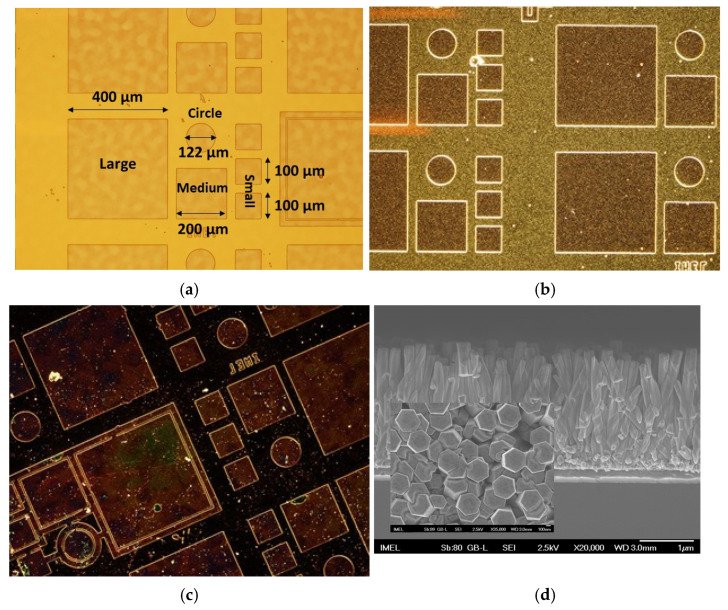

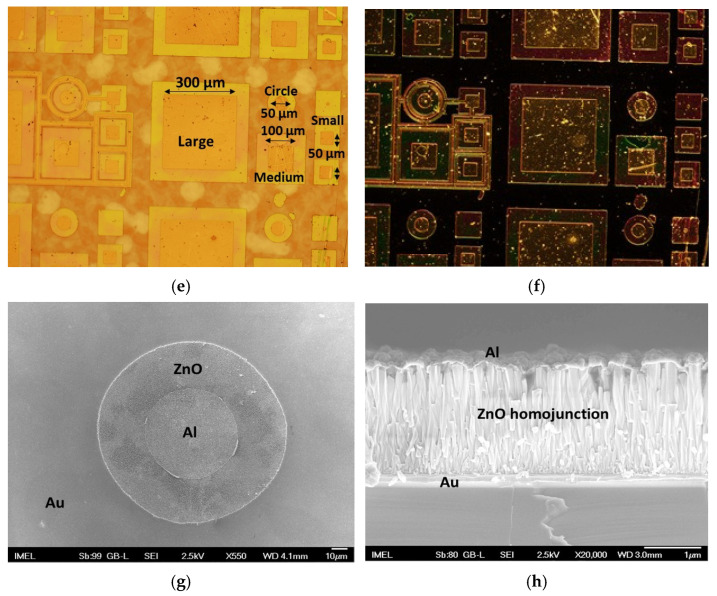
Representative optical microscope images (magnification ×10) of the device patterns after the hydrothermal growth of the Li:ZnO NR layer: (**a**) bright field and (**b**) dark-field image. (**c**) Optical microscope image (magnification ×10; dark field) and (**d**) SEM cross-sectional image (scale bar: 1 μm) after the 2nd growth step of the undoped ZnO nanotextured layer on top of the Li:ZnO NRs for the formation of the chemically produced homojunction. Inset in (**d**): top view of the homojunction demonstrating the coalescence of the u-ZnO NRs and the formation of the nanotextured film on top of which the top contact will be formed (scale bar: 100 nm). Optical microscope images (magnification ×10) after the deposition of the top Al electrode: (**e**) bright field and (**f**) dark field. SEM images after the deposition of the top Al electrode: (**g**) top view (scale bar: 10 μm) and (**h**) cross section (scale bar: 1 μm).

Four types of devices (Table 1) were realized on 3” Si (100) substrates (Siegert Wafer, Aachen, Germany) according to the following process, schematically depicted in Figure 2:

(1) A 100 nm Au layer was deposited via magnetron sputtering, serving as a common bottom electrode for all devices.
(2) A ZnO seeding layer was deposited via spin-coating a 40 mM Zinc Acetate Dihydrate Zn(CH_3_COO)_2_·2H_2_O, Sigma-Aldrich, Darmstadt, Germany) solution in ethanol (Carlo Ebra, Cornaredo, Italy) at 1000 rpm for 30 s, followed by a 10 min annealing at 500 °C on a hot plate. This spin-coating and annealing step was repeated 10 times, as detailed in previous studies [15,27,28,29,30,33].
(3) Positive-tone optical lithography was performed to selectively grow ZnO nanostructures within the defined patterns. These patterns on each die included circles with diameters of 112 μm, as well as squares with side lengths of 100 μm, 200 μm, and 400 μm (Figure 3a,b). Based on their shapes and sizes, the devices will be hereafter referred to as “circles” for the circular devices and as “small”, “medium”, and “large” for the square ones.
(4) After lithography, the wafers were immersed face-down in separate equimolar solutions of zinc nitrate hexahydrate (Zn(NO_3_)_2_·6H_2_O, Sigma-Aldrich, Darmstadt, Germany) and hexamethylenetetramine (C_6_H_12_N_4_, HMTA, Panreac, Barcelona, Spain) with varying concentrations of lithium nitrate (LiNO_3_, Fisher Chemical, Pittsburgh, PA, USA) for 2 h in order to produce ZnO nanorods of varying concentrations of Li doping (Figure 3a,b). These nanorods constituted the nominally “p-type” layer of the homojunctions [26,33,34,35]. The molar ratio of the lithium salt to the zinc salt precursor determined the doping level (see Table 1 for sample conditions and naming). The growth was conducted at 87 °C in a water bath.
(5) Following the growth step, the samples were thoroughly rinsed with DI water (without removing the photoresist) and immersed in a fresh 200 mM equimolar solution of zinc nitrate hexahydrate (Zn(NO_3_)_2_·6H_2_O, Sigma-Aldrich) and HMTA for 2 h to form the nominally “n-type” layer of the homojunction [36]. The solution was maintained at 87 °C in a water bath throughout this growth step. This way, what we have termed a nanotextured film is produced, resulting from the coalescence of the fast-growing nanorods [27,36]. This approach enables the formation of a suitable layer on top of which the top contact of the junction may be directly formed without the need to fill the “pores” with other materials for passivation, such as PDMS or PVDF, and additional fabrication steps as in previously reported works (e.g., [37,38]). Moreover, no additional doping was explored/used in this layer based on the established fact that ZnO is inherently an n-type semiconductor due to the presence of point defects [36,39,40].
(6) Afterwards, a lift-off step was performed, followed by what is depicted in Figure 3c,d.
(7) A second positive-tone lithographic step was performed in order to form the top electrodes.
(8) One hundred nm of Al was deposited via e-beam evaporation to form the top electrodes. The diameter of the electrode for the circular device was 50 μm, and for the “small”, “medium”, and “large” square devices, the square sides were 50, 100, and 300 μm, respectively (Figure 3e–g).
(9) Finally, a lift-off step concluded the fabrication process flow (Figure 3g,h).

### 2.4. Electrical Characterization

The electrical characterization of all devices (commercial diodes and the ZnO-based devices) was conducted with a Keithley 4200-SCS (Semiconductor Characterization System; Tektronix, Beaverton, OR, USA) comprising the Remote Pre-Amp option for low current measurements, and SUSS MicroTec Probes (Munchen, Germany) were used. Built-in measuring software provided with the system (KITE, V8.2), comprising the full development option, was used to record the data, allowing the modification of the programming codes. The range of the measurements was limited by the maximum current (0.1 A) provided by the voltage source or the power limitation of the devices.

## 3. Results and Discussion

### 3.1. Proof of Concept Using Commercial Diodes

**Figure 4:**
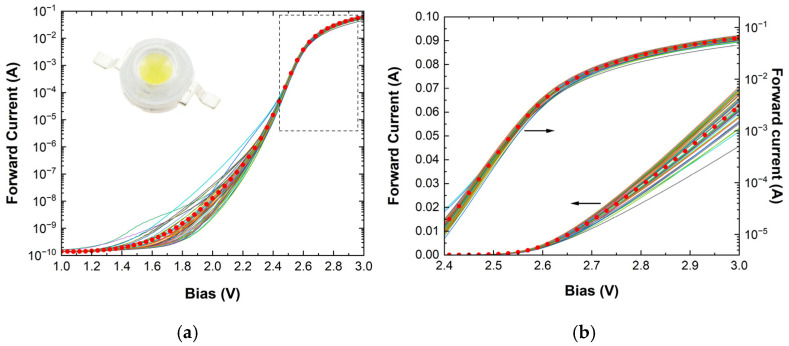
Forward I-V characteristics of the 49 white LEDs in (**a**) a semi-log plot and (**b**) both linear and semi-log plots for the high-bias range (dashed box in (**a**)). Red circles depict the average behavior.

Initially, the use of commercial diodes (LEDs) to construct a PUF was examined in order to test the feasibility of employing diodes as the basic building block of a PUF. Only the forward current-voltage (IV) characteristics of the diodes were exploited. A typical dispersion in the I-Vs measured for 49 similar white LEDs is shown in Figure 4, where the average behavior is also depicted (circle symbols). It can be seen that the LEDs have sufficient variability only in a limited region of data. Three areas (bias ranges) were analyzed and used for comparison: the low voltage area (below 2.2 V), the exponential region (2.4 to 2.6 V), and the higher voltage region where the series resistance dominates. The correlation matrix (Appendix A) among all the extracted parameters of the entire ensemble revealed that a subset of more than twelve independent parameters has to be used to efficiently distinguish each individual diode, while ten were marginally sufficient. These parameters include, in addition to the ideality factor and the saturation current, currents, and series resistances at specific biases. For characteristics exhibiting higher variability, a smaller set of “comparison parameters” may become adequate.

**Figure 5:**
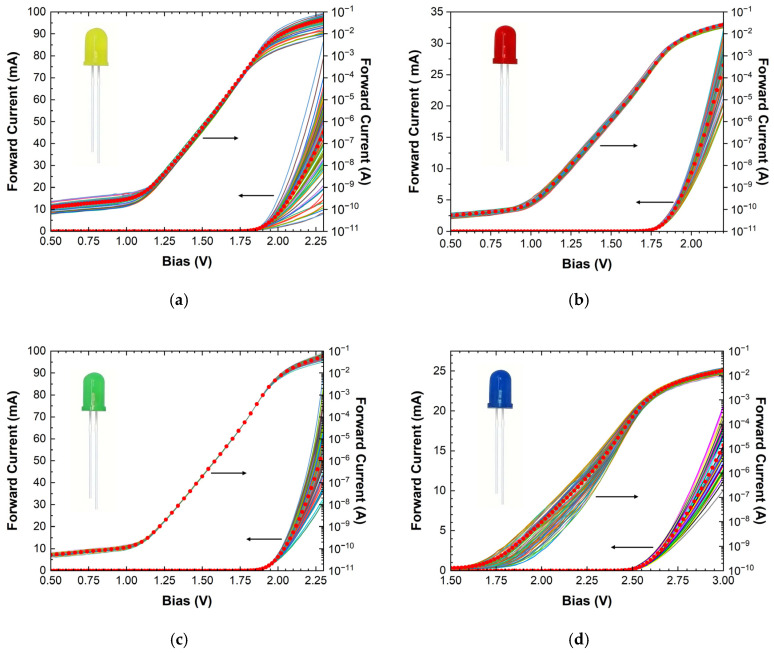
Forward I-V characteristics in both linear and semi-log plots of a hundred (**a**) yellow, (**b**) red, (**c**) green, and (**d**) blue LEDs. The red circles in all graphs depict the average behavior.

Different types of diodes can exhibit different variability, as demonstrated in Figure 5. In the case of yellow LEDs (Figure 5a), the exploitable variability was mainly found in the ohmic region, while the exponential behavior of all specimens was almost identical. Therefore, parameters such as the ideality factor and saturation current could not be used for the identification procedure. The red LEDs exhibited a more “uniform” dispersion (Figure 5b), promoting them to potential PUF candidates. The green LEDs (Figure 5c) exhibited similar statistical variability to that of the yellow LEDs in their IVs, but with considerably smaller dispersion, rendering their applicability to PUFs less likely. Blue LEDs (Figure 5d) displayed a behavior very similar to that of white LEDs, meaning that only one of them (white or blue) should be used in a practical embodiment of an LED-based PUF authentication system to avoid unnecessary complexity. These results indicate that the procedure for obtaining the digital implementation of the PUF, by combining different types of diodes (or LEDs), though feasible in principle, should follow a detailed study of each available technology (manufacturer) and a careful selection of the most appropriate variables as comparison criteria. In addition, a considerably large number of variables (at least 10) seems to be necessary to identify each device (as derived from the corresponding correlation matrices), which renders the construction of PUFs rather complex. One could argue that distinguishing a device among many similar ones could be significantly improved if the “size” factor were incorporated into the device’s construction. However, this is not always feasible with off-the-shelf components that have fixed designs. Since size-dependent phenomena could greatly enhance differentiation among similar devices, a preferable approach may be to design customizable devices with inherent variability, where their operation is largely dependent on their size. This idea led to the exploration of “flawed” devices with suboptimal performance, featuring nanostructures with stochastic morphology and size dispersion, which introduce inherent variability. This is examined and analyzed in the following sub-section.

### 3.2. ZnO Homojunctions as Eco-Friendly PUF Generators

**Figure 6:**
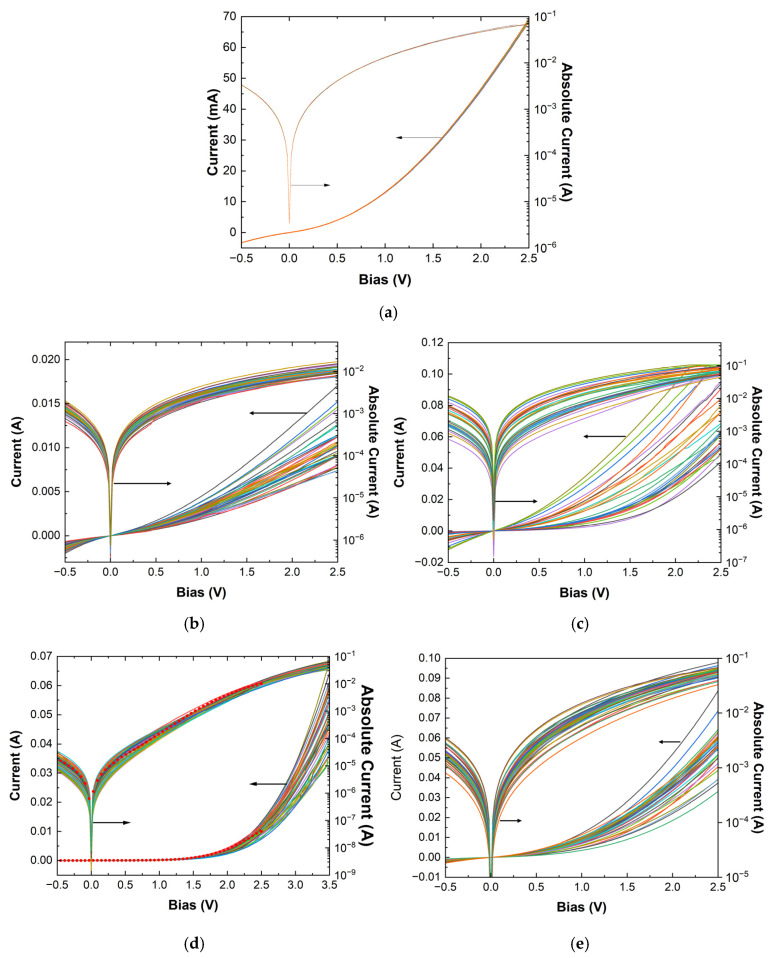
(**a**) I-V characteristics of a randomly selected medium-size device from the RU-0.5 wafer were repeatedle measured eight times, and heerin presented in a linear and semi-log plot. Differences are not discernible even at the semi-log plot. I-V characteristics both in linear and semi-log plots of 40 medium-size devices from wafers (**b**) RU-0, (**c**) RU-0.5, (**d**) RU-1.0, and (**e**) RU-2.0. Red circles in (**d**) depict the I-V characteristic of a random medium-size device of RU-1.0 measured over 7 years ago when the devices were fabricated.

In this study, the main concept was to use chemically produced ZnO nanostructured homojunctions as authentication elements, capitalizing on their inherent variations induced via chemical doping with different concentrations of Li and the variations arising from the stochastic nature of the chemically grown nanostructures in terms of morphology and size distribution. Building on this premise, it was critical to assess whether the inherent variability can coexist with the stability and repeatability required for a robust PUF generator. To that end, the first steps involved evaluating the consistency of the electrical behavior of the ZnO homojunctions over time. To minimize any size-related effects (which will be discussed later), the study focused on medium-sized devices with a 100 × 100 μm^2^ contact area (Figure 3a,e). Figure 6a presents repeated measurements of a medium-sized device from the sample RU-0.5, selected randomly from the entire 3” wafer and demonstrating the stability of individual devices. Subsequently, after the current-voltage characteristics of 40 devices across all samples were analyzed, it was observed that, in over 90% of cases, each device’s current measurements remained self-consistent to at least three digits of accuracy, as was the case of the device shown in Figure 6a. In terms of robustness, notably, the devices demonstrated exceptional stability over time. As shown in Figure 6d, the red circles represent initial measurements from a medium-sized device of sample RU-0.5, taken over seven years ago when the devices were first fabricated. This long-term stability is particularly remarkable, given that the devices lack a passivation or insulating layer—commonly used for such devices—and have been stored in ambient conditions outside a clean-room environment without any special protective packaging until their recent reuse as potential PUF generators.

As can be seen in Figure 6b–e, the devices exhibit significant variability, which can be leveraged to create a unique identification system. Although they perform poorly as diodes—deviating substantially from the expected behavior of an ideal diode due to incomplete p-type conversion [32], which more likely causes their operation to resemble an n^−^/n homojunction—this flaw becomes a key advantage for PUFs.

Upon closer inspection, the general I-V behavior of devices RU-0, RU-0.5, and RU-2.0 is quite similar, with RU-2.0 standing slightly out due to the continuous bending of the I-V characteristic (see semi-log plot in Figure 6e), which makes the comparison of currents even in the low bias region easier. RU-1.0, on the other hand, demonstrates a more consistent behavior across the wafer (reduced variability); however, its I-V characteristics are such that a larger number of distinguishable parameters can be extracted.

Both of these types of behaviors are useful in order to randomly mix into an array of devices and enhance the individual overall electrical behavior of the ensemble. Even defective devices—acting as resistors—could be used, in conjunction, to enhance variability and further increase the possible combinations, producing more distinguishable identification units.

**Figure 7:**
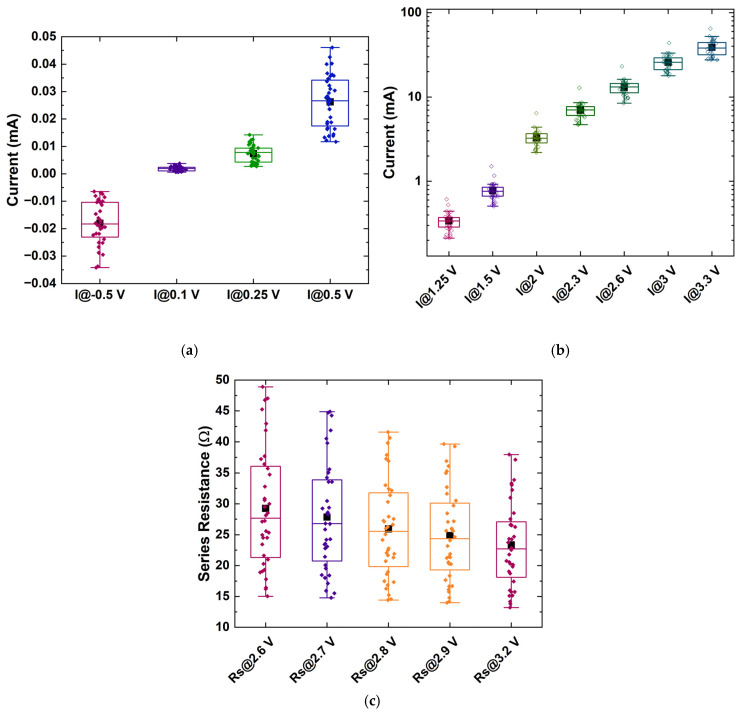
Distribution of (**a**) currents at a reverse bias of −0.5 V and within the low-bias regime, (**b**) currents within the high-bias regime for sample RU-1.0, and (**c**) series resistances at various voltages within the high-bias regime. The rectangular boxes indicate the values between 25% and 75% of the maximum obtained value. Also, a few outliers can be observed. Black squares indicate the mean value, while horizontal lines within the boxes mark the median value.

**Figure 8:**
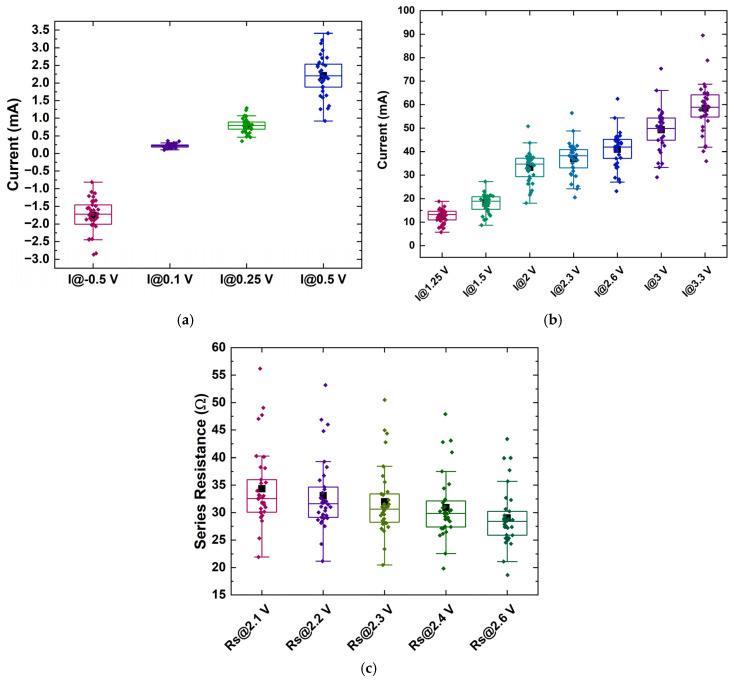
Distribution of currents (**a**) at a reverse bias of −0.5 V and within the low-bias regime and (**b**) within the high-bias regime for sample RU-2.0, and (**c**) distribution of the series resistance. The rectangular boxes indicate the values between 25% and 75% of the maximum obtained value. Black squares indicate the mean value, while horizontal lines within the boxes mark the median value.

**Figure 9:**
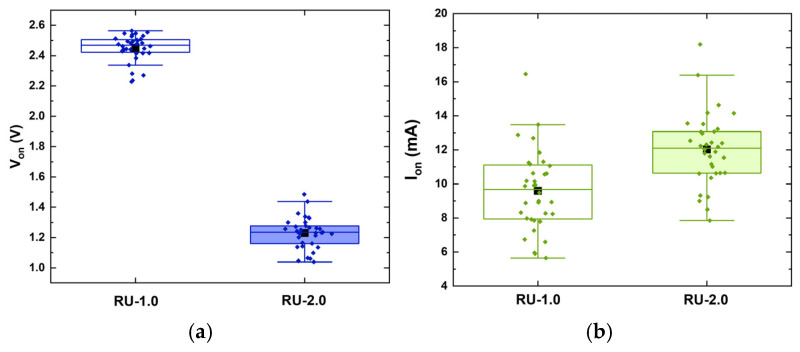

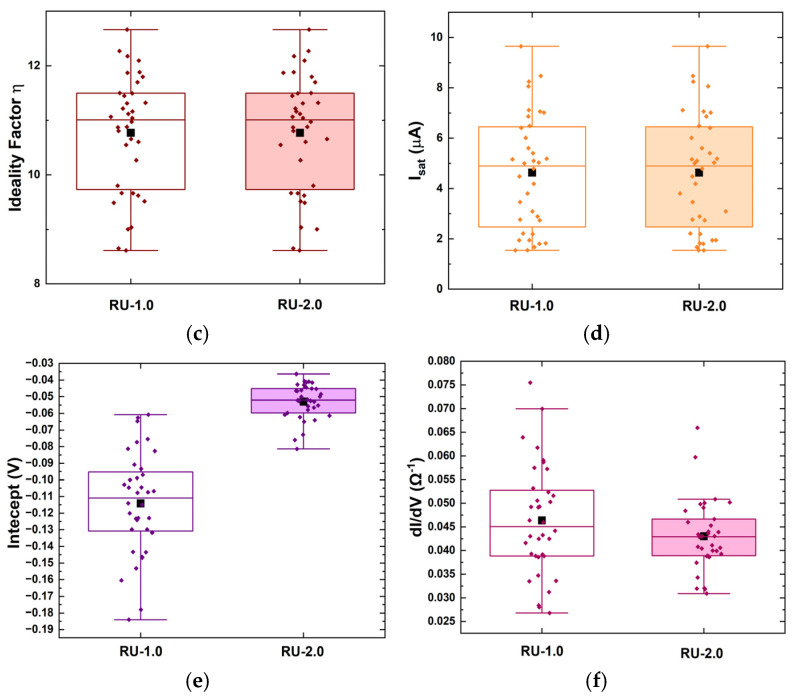
Distribution of (**a**) the turn-on voltage *V_on_*, (**b**) the current at *V_on_*, *I_on_*, (**c**) the ideality factor *η*, (**d**) the saturation current *I_s_*, (**e**) the intercept, and (**f**) the slope of the linear region *dI*/*dV* of both RU-1.0 (clear rectangles) and RU-2.0 samples (shaded rectangles). The rectangular boxes indicate the values between 25% and 75% of the maximum obtained value. Black squares indicate the mean value, while horizontal lines within the boxes mark the median value.

**Figure 10:**
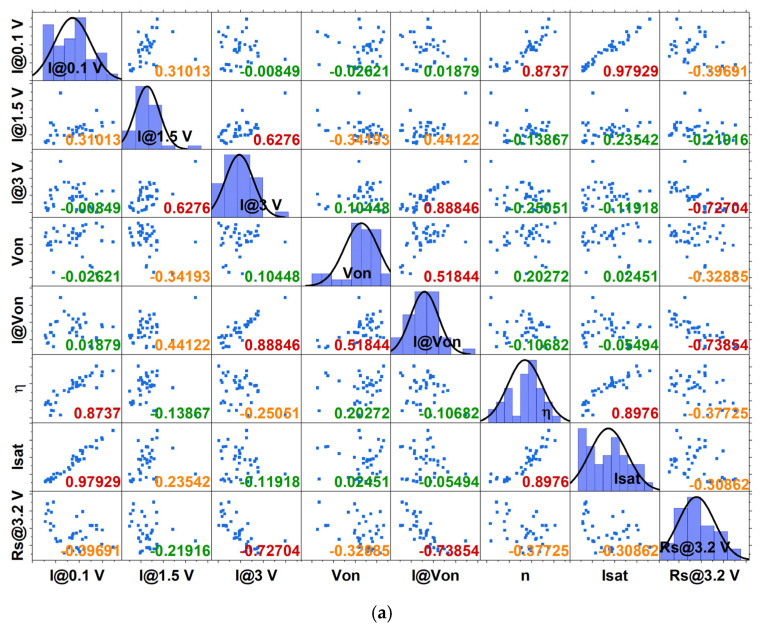

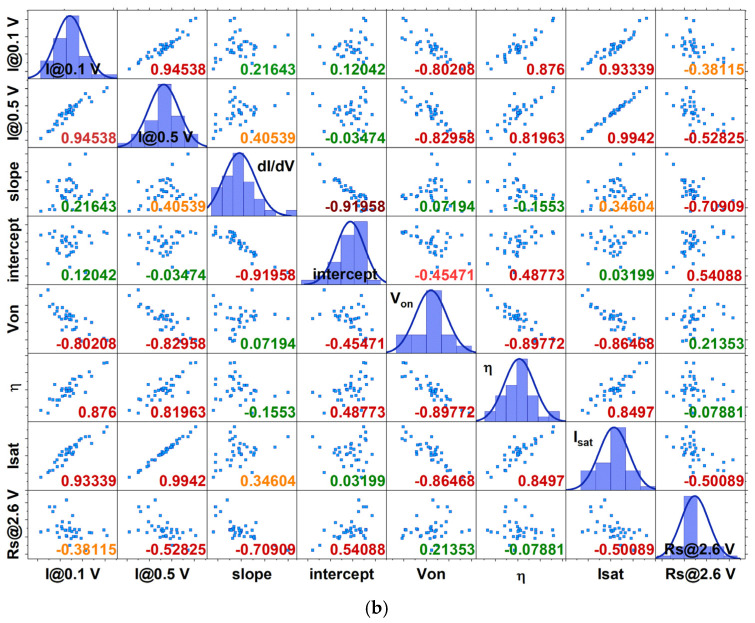
Visualization of the correlation matrix by including the scattering plot between any two parameters selected for the final subset for identification: (**a**) RU-1.0 and (**b**) RU-2.0 samples. The upscaling gradual color code of the correlation coefficient is green (r < 0.3), orange (0.3–0.4), and red (>0.45). In the diagonal (where r = 1), the graphs depict the distribution of each parameter’s values, along with the extracted normal distribution curve.

As a result, in order to demonstrate the feasibility of using cost-efficient (yet sub-optimal) diodes as robust PUF generators, RU-1.0 and RU-2.0 were deemed sufficient for identification, and only these two were further analyzed. Following the general concept analyzed in Section 2.1, about 20 parameters were extracted from their IVs, comprising currents at specific biases *I*(*V_i_*), the ideality factor *η*, the saturation current (*I_sat_*), the turn-on voltage (*V_on_*), the current at turn-on voltage (*I_on_*), the slope (*dI/dV*), and the intercept in the linear region (high-bias regions), as well as the series resistance at various biases *R_s_*(*V_i_*) (Figure 7, Figure 8 and Figure 9), while the correlation matrices were calculated and visualized in Figure 10.

As expected from the I-V plots, in the case of the RU-1.0 devices, low currents -for biases up to 1.25 V—have similar behavior, including the reverse bias currents (Figure 7a). This was further verified using the correlation coefficient between any two of them (Appendix A). Thus, from this region of bias, only one current can be exploited as a unique identifier, while the rest can be considered redundant. Currents at higher biases tend also to exhibit similar behavior to each other (Figure 7b), having a high correlation (Figure 10a); thus, one value of current at this region can again be useful, and the same stands for the series resistances (Figure 7c). Also, *V_on_* can be extracted from the currents measured above 3 V (Figure 9a), as well as the current at *V_on_*, *I_on_*, Figure 9b), while the ideality factor and the saturation current can be extracted from the values around 1 or 2 V (Figure 9c,d, respectively). Finally, it was seen that the intercept and the slope exhibited high variability (Figure 9e,f, respectively) and could be employed as identifiers. Overall, one needs to choose parameters (the identification parameter set) that have as low correlations as possible to each other in order to constitute the identity of each sample.

A similar study for the RU-2.0 device statistics was performed, and the results are summarized in an analogous way in Figure 8 and Figure 9. Following the same method–procedure, it was observed that currents are more dispersed (Figure 8a,b) but also more correlated than in the RU-1.0 homojunctions case. The series resistance, as in the case of RU-1.0, demonstrated the same degree of variability (Figure 8c) and high correlation (Appendix A), and only one can be selected as a unique identifier. As a result, two or even one current (at a specific bias) can be selected for comparison, most preferable at low voltages. The rest of the parameters selected for the final comparison were *V_on_*, the slope in high biases, the ideality factor *η*, and series resistance (at high bias).

After this initial assessment, a subset of parameters exhibiting the highest variability without evident redundancy was examined for their correlation factors in both devices. The correlation factors and any “hidden” correlations (or, more accurately, the desirable lack thereof) are summarized and demonstrated concisely in Figure 10a,b. Parameters with low correlations (r < 0.3) are highlighted in green, with medium correlations in orange and strong correlations in red. As shown in Figure 10 and detailed in Appendix A, from the initial 22 parameters for RU-1.0 and 17 for RU-2.0, a subset of 8 parameters for each device suffices to generate unique identifiers—a notable reduction from the 12 parameters required for commercial diodes. Considering that these devices exhibit variations in current density relative to their size, one can obtain 3 × 8 = 24 unique identifiers per sample, as the circles and small devices from each die have practically the same effective area and exhibit nearly identical behavior.

As a final step towards generating robust PUFs, the following was taken into consideration. Since each parameter exhibits a different dispersion, expressed through their mean value and standard deviation, a standardized approach was required. Initially, comparisons were made using confidence limits defined as a percentage of each parameter’s value. However, this method required setting a unique percentage for each parameter, complicating the recognition process. A more straightforward approach was adopted by defining recognition limits in terms of standard deviations, allowing a single factor, *f*, to be applied uniformly across all parameters. The recognition limits were thus defined as *k_i_* − *f* × *σ* and *k_i_* + *f* × *σ*, where a tested value falling within this range was considered a successful recognition. For instance, setting *f* = 1 meant that any device with a parameter within *k_i_* − *σ* and *k_i_* + *σ* passed the recognition test.

To evaluate the method, the recognition of the “average device”—expected to be the most challenging—was initially tested for RU-1.0. Identification required all selected parameters of a device to satisfy the recognition criteria. With *f* = 1, seven additional devices were identified as similar. Reducing *f* to 0.7 decreased this number to five, *f* = 0.5 resulted in two similar devices, and *f* = 0.3 allowed the recognition of only the original device. The optimal value of *f* for the average device was found to be 0.39, meaning that, for *f* ≤ 0.39, only the original device was identified. When this procedure was repeated for all devices, it was concluded that setting *f* = 0.36 or lower ensured the recognition of only the original device, while the average optimal *f* across all tested devices was 0.60.

Despite optimization, it remains possible for two or more devices to be nearly identical and indistinguishable unless a sufficiently small *f* is chosen. However, rather than enforcing overly strict criteria, a more effective approach is to group multiple devices and require all to pass the identification test. This method forms a “matrix” of devices, improving recognition efficiency and minimizing failure rates.

For RU-2.0 samples, setting *f* < 0.33 ensured accurate recognition of all devices, with an average optimal *f* of 0.65. Therefore, by incorporating RU homojunction devices and integrating different technologies, such as RU-1.0 and RU-2.0, the substantial variability among devices can be exploited. Choosing *f* ≈ 0.6 allows the formation of a unit with a unique electrical signature. ZnO homojunction diodes offer additional advantages, including increased variability, diverse electrical behaviors that can be combined, and the ability to fabricate devices of different sizes and shapes within the same die or in the form of matrix addressable devices of varying sizes and Li doping. Furthermore, the ecological benefits and low cost of the material present a promising alternative to conventional, expensive PUFs, offering a low failure rate and potential for further optimization.

To quantify the potential of diode-based PUFs prior to digital implementation, we conducted an a priori statistical analysis of diode parameters to assess the effectiveness of our approach and guide further optimizations. This preliminary evaluation leverages the inherent variability in diode electrical characteristics to estimate key PUF metrics, such as entropy, uniqueness, and uniformity, before constructing the digital PUF.

Initial measurements using a batch of nominally identical diodes revealed significant variability, with all extracted electrical parameters exhibiting approximately normal distributions, consistent with prior studies on semiconductor manufacturing variability [41,42]. The parameters, including currents at various voltages (such as $I@0.1V,I@1.5V,I@3V,I@V_{\mathrm{on}},I_{\mathrm{sat}}$), turn-on voltage ($V_{\mathrm{on}}$), ideality factor (n), and series resistance ($R_{s}@3.2V$), are potential candidates for PUF construction. By utilizing a broader parameter set (n=8), we enhance the entropy potential per diode compared to single-parameter methods, which often rely solely on threshold voltage or current or even a delay time [31,42,43], to extract only 1-bit for each parameter.

The inputs for our subsequent statistical analysis include the mean values ($\mu_{i}$) and standard deviations ($\sigma_{i}$) of the n=8 parameters, along with their correlation matrix (R). To maximize entropy, we select a subset of parameters with minimal correlation, ensuring near-independence. A case study based on our measurements, as described earlier, and the correlation matrix R (Appendix A) with eigenvalues [3.53, 1.95, 0.92, 0.54, 0.45, 0.36, 0.22, 0.03], yields an effective number of independent parameters ($n_{\mathrm{eff}}\approx 5.92$), calculated as $n_{\mathrm{eff}}={\left(\sum \sqrt{\lambda_{i}}\right)}^{2}/\sum \lambda_{i}$, where $\lambda_{i}$ are the eigenvalues of R. This $n_{\mathrm{eff}}$ is used in subsequent entropy and uniqueness calculations.

To further increase entropy, we introduce multi-level digitization, quantizing each parameter into q=10 levels with a recognition margin defined as $f\cdot \sigma_{i}$, where f=0.6 is optimized to balance entropy and reliability, after examining our results and mimicking the recognition procedure. This approach departs from traditional binary digitization, capturing finer variations in parameter values. Previous studies on multi-level quantization in PUFs [44] have demonstrated that finer quantization enhances entropy by exploiting more of the available variability; a value of q=10 will offer a practical trade-off between entropy and noise sensitivity. The choice of f=0.6 minimizes noise-induced errors in an optimistic (best-case) scenario where noise is initially neglected to estimate the maximum entropy. Future analyses will incorporate noise as an additive term to the parameter variances.

Following parameter extraction and statistical analysis, we adopt f=0.6 and q=10 for digitization in the PUF construction. Challenge–response pairs (CRPs) are generated by selecting subsets of parameters as challenges and mapping responses to quantized levels. The entropy per parameter ($H_{i}$) is computed based on the quantization levels, accounting for the probability distribution of q=10 symbols and yielding $H_{i}\approx 3.32$ bits per parameter after adjusting for bit-error rate effects. The total entropy for m diodes is given by $H_{\mathrm{total}}=m\cdot n_{\mathrm{eff}}\cdot H_{i}$. For m=25 diodes, $n_{\mathrm{eff}}\approx 5.92$, and $H_{i}\approx 3.32$ bits, the total entropy is approximately 491.50 bits, significantly exceeding the 128 or 256 bits typically required for cryptographic applications. For a smaller array of m=16 diodes (e.g., a 4 × 4 grid), the entropy is approximately 314 bits, still sufficient for most security applications.

This approach is inherently scalable, allowing entropy to be increased by incorporating more diodes (m) or parameters (n) or both. We must also mention that, if we use all parameters (22 in RU-1.0), the effectively independent variables are $n_{\mathrm{eff}}\approx 12.5$, which gives us an upper limit. Choosing a smaller subset of 8–10 is close to this limit, keeping the PUF modeling more simple and effective. Moreover, choosing a proper subset of parameters maximizes efficiency in terms of the entropy per device (*H*_diode_/*n*) and also minimizes the measurement cost. The use of multi-level quantization and a broad parameter set positions diode-based PUFs as a promising candidate for high-entropy, low-cost security primitives, with ongoing work focusing on the digital construction of the PUF and experimental validation.

## 4. Conclusions

This comprehensive study has delved into the feasibility of utilizing chemically synthesized ZnO nanostructured homojunctions as physical unclonable function (PUF) elements, aiming to enhance security in electronic systems. The research focused on leveraging the intrinsic variability introduced through lithium (Li) doping and the stochastic nature of hydrothermal growth processes.

A thorough analysis of the electrical characteristics (IVs) of these ZnO-based devices was conducted, leading to the identification of key parameters exhibiting significant variability, stability, and reproducibility. By meticulously selecting a minimal subset of these parameters, we ensured reliable device recognition, thereby demonstrating the potential of ZnO-based devices as cost-effective and sustainable alternatives to traditional PUFs.

The study also highlighted the advantages of ZnO homojunction diodes, including increased parameter diversity and enhanced security potential. The adoption of a standardized recognition approach, grounded in statistical variations, confirmed the feasibility of these devices as robust and eco-friendly PUF generators.

However, it is important to acknowledge certain limitations within this study. The long-term stability of these devices under varying environmental conditions remains to be thoroughly investigated (e.g., temperature, humidity, the presence of gases, etc.). Additionally, the scalability of the fabrication process for mass production warrants further exploration.

Future research should focus on addressing these limitations by examining the durability of ZnO-based PUFs in diverse environmental settings and optimizing fabrication techniques for large-scale production. Moreover, integrating ZnO-based PUFs into existing authentication frameworks and exploring their applicability in emerging technologies could provide valuable insights. In addition, future work can be the addition of devices with different sizes (cross-section area) and shapes that, due to the inherent flaws and stochastic nature of hydrothermal growth, may affect the electrical behavior. Also, more parameters can be included in the evaluation and distinction of the devices, such as electrical noise, capacitances in different biases, and the breakdown voltage and current of the devices. This preliminary work nonetheless indicates that such additions are not necessary but could probably improve the overall recognition ability.

In conclusion, ZnO homojunction diodes present a promising and eco-friendly solution for hardware-based security applications. Their integration into scalable authentication frameworks holds significant potential, contributing to the advancement of low-cost, highly secure, and environmentally sustainable digital identity solutions.

## Figures and Tables

**Table 1:** Names of the 4 types of devices and the “p-layer” hydrothermal growth parameters.

Sample Name	Zinc Nitrate Hexahydrate Concentration (mM)	HMTA Concentration (mM)	Lithium Nitrate Concentration (mM)	Lithium-to-Zinc Molar Ratio
RU-0	40	40	0	0
RU-0.5	40	40	20	0.5
RU-1.0	40	40	40	1.0
RU-2.0	40	40	80	2.0

## Data Availability

The raw data supporting the conclusions of this article will be made available by the authors upon request.

## Appendix A

A full correlation matrix was construed from 21 selected parameters, resulting into a 21 × 21 matrix for the commercial white LEDs. Each row of the matrix includes, apart from the calculated value of the correlation coefficient, the correlation graph between all pairs of parameters to better demonstrate the method and the usefulness of the matrix to readily indicate the parameters with the lowest correlation among them.

Full correlation matrices for RU-1.0 (22 × 22 parameters) and RU-2.0 (17 × 17 parameters) for all parameters examined in this study, where the redundancy of the currents and series resistance is obvious. From their inspection, the final sub-set of eight parameters was selected (as described in Figure 10a,b).
